# Relationship of the metabolic score for insulin resistance and the risk of stroke in patients with hypertension: A cohort study

**DOI:** 10.3389/fendo.2022.1049211

**Published:** 2022-12-05

**Authors:** Xintian Cai, Junli Hu, Qing Zhu, Mengru Wang, Shasha Liu, Yujie Dang, Jing Hong, Nanfang Li

**Affiliations:** Hypertension Center, Xinjiang Hypertension Institute, NHC Key Laboratory of Hypertension Clinical Research, Key Laboratory of Xinjiang Uygur Autonomous Region, Xinjiang Clinical Medical Research Center for Hypertension Diseases, People’s Hospital of Xinjiang Uygur Autonomous Region, Urumqi, Xinjiang, China

**Keywords:** metabolic score for insulin resistance, hypertension, stroke, ischemic stroke, cohort study

## Abstract

**Background:**

The current status of the dose-response relationship between the metabolic score for insulin resistance (METS-IR) and new-onset stroke in hypertensive patients and its subtypes is unclear. This study aimed to determine the association between METS-IR and incident stroke and its subtypes within a cohort of Chinese hypertensive patients.

**Methods:**

A total of 14032 hospitalized patients with hypertension from January 1, 2010, to December 31, 2021, were included in this retrospective cohort study. Cox models and restricted cubic splines were applied to determine the association between METS-IR and the risk of stroke.

**Results:**

During a median follow-up of 4.80 years, 1067 incident stroke cases occurred. Patients in the highest quartile group of METS-IR levels exhibited a higher risk of stroke (HR, 1.80; 95% CI, 1.50-2.17) and ischemic stroke (HR, 1.96; 95% CI, 1.60–2.42) than those in the lowest quartile group. However, no significant associations were observed between METS-IR and the risk of hemorrhagic stroke. Restricted cubic spline analysis suggested a nearly J-shaped association between METS-IR and risk of stroke and ischemic stroke (P for nonlinearity < 0.001). METS-IR did produce a significant improvement in the C statistic when added to the basic model (from 0.637 to 0.664, P < 0.001). Notably, the addition of METS-IR to the basic model resulted in a significant improvement in predicting incident total stroke and ischemic stroke.

**Conclusions:**

This cohort study suggests a relationship between METS-IR and the risk of stroke and ischemic stroke. Further studies are required to elucidate the underlying mechanisms.

## Introduction

Stroke has developed into a significant global health problem (1–3). According to the latest annual report in 2019, there are currently more than 20 million stroke patients in China (4). Available evidence suggests that hypertension is the most significant risk factor for stroke (5, 6). Therefore, identifying hypertensive patients with a high risk of stroke is clinically essential to improve risk stratification.

Abnormalities in glucose and lipid metabolism are common in hypertensive patients, and insulin resistance (IR) serves an essential function in this biological procedure (7–9). IR is not only an important contributor to the progression of arterial stiffness, endothelial dysfunction, and metabolic syndrome but also a risk factor for stroke development. Therefore, early discovery and control of IR may help in the early prevention of stroke (9–12). Currently, there are several methods available to assess IR. First, in the 1970s, the euglycaemic-hyperinsulinaemic clamp (EHC) was proposed as the gold standard for the assessment of IR (13). However, this method is challenging to apply in large-scale clinical and epidemiological studies due to its drawbacks such as complexity, cost, and invasiveness (14). Secondly, given its accessibility and low cost, the triglyceride glucose index (TyG), which is generated from fasting blood glucose (FPG) and fasting triglycerides (TG), is presently the most widely used marker of IR (15). Nevertheless, the index only includes two metabolic variables and does not take into account how diet and cholesterol affect cardiovascular disease. As a result, TyG might not accurately depict how IR affects the cardiovascular system (16). Fortunately, a novel IR marker called the metabolic score for IR (METS-IR) has just been created by Bello-Chavolla et al. (17). The most potent IR measure outside of EHC, METS-IR, combines FPG, TG, high-density lipoprotein cholesterol (HDL-C), and body mass index (BMI), which represents nutritional status (18, 19). So far, numerous studies have found METS-IR to be associated with various cardiometabolic diseases, including hypertension, diabetes, non-alcoholic fatty liver disease, and ischemic heart disease (18, 20–22). Therefore, METS-IR may be clinically important for risk stratification of new-onset stroke in hypertensive patients. In addition, the current status of the dose-response relationship between METS-IR and new-onset stroke in hypertensive patients and its subtypes is unclear.

In this study, we sought to determine the association between baseline METS-IR and stroke and its subtypes among Chinese hypertensive patients.

## Material and methods

### Study population

**Figure 1:**
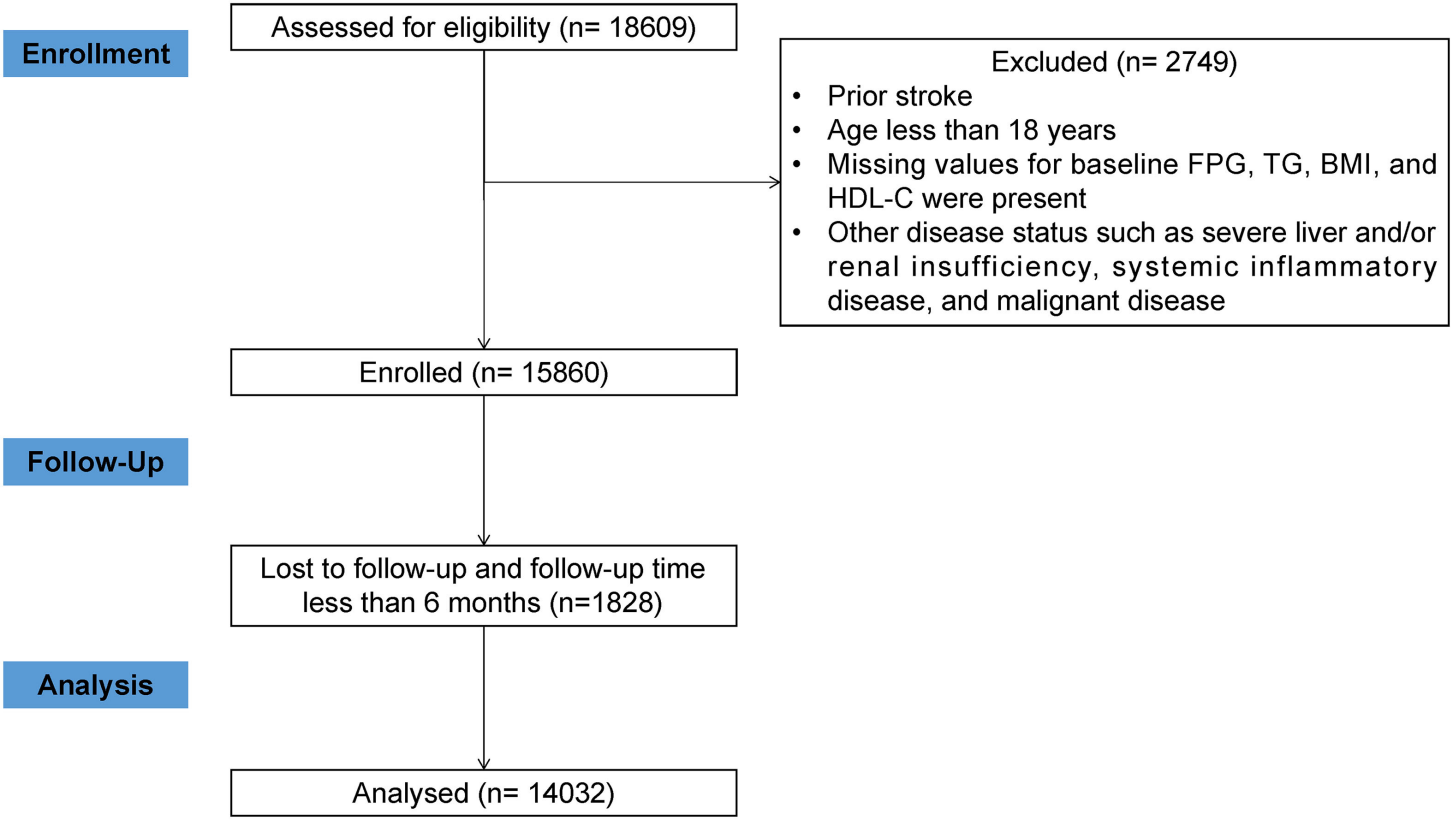
A study flowchart.

We conducted a cohort study of hypertensive patients followed at a hypertension center (the People’s Hospital of Xinjiang Uygur Autonomous Region). Deidentified patient data retrieved from electronic medical records was used, including the date of birth, sex, physical measurements, diagnostic codes according to the International Classification of Diseases, 10th Revision (ICD-10), medication prescriptions, and laboratory results. A total of 18609 patients with hypertension were recruited from January 1, 2010, to December 31, 2021. After strict exclusion criteria, a total of 14032 patients were included (**Figure 1**). A comparison of baseline characteristics of participants included and excluded from this study may be found in **Table S1**. The ethics application was approved by the Ethics Committee of the People’s Hospital of Xinjiang Uygur Autonomous Region. Informed consent was waived owing to the retrospective nature of the study. Moreover, this study followed STROBE reporting guidelines.

### Data collection and definitions

The information provided by the electronic medical record includes demographic data, lifestyle factors, laboratory measurements, medical history, and medication history. BMI was calculated as body weight (in kilograms) divided by height (in meters squared). Blood pressure (BP) and heart rate were measured by standard procedures. Smoking status was categorized as non-smokers and current smokers. Alcohol consumption status was divided into non-drinkers and current drinkers. Blood samples were collected after an overnight fast. Fasting plasma glucose (FPG), total cholesterol (TC), triglycerides (TG), high-density lipoprotein cholesterol (HDL-C), low-density lipoprotein cholesterol (LDL-C), high-sensitivity C-reactive protein (hsCRP), hemoglobin A1c (HbA1c), uric acid (UA), and cystatin C (Cys C) were measured. The estimated glomerular filtration rate (eGFR) was calculated using the CKD-EPI equation. The participants’ prior medical histories were evaluated using ICD-10 codes. To ensure the accuracy of diagnoses, diabetes (E10-E14) and dyslipidemia (E78) were regarded as present if a participant was treated ≥ 2 times. Coronary heart disease (CHD) (I24 and I25) was considered present if a participant was treated ≥ 1 time. The Charlson Comorbidity Index (CCI) was determined from claims data during a lookback period of 2 years before the baseline. The CCI (using ICD-10 codes) was calculated, as reported previously (23). The list of medications included in the study is available in **Table S2**. METS-IR was calculated as previously reported, and is presented as follows: METS-IR = Ln[(2 × FPG (mg/dL))+TG (mg/dL)] × BMI (kg/m^2^))/(Ln[HDL-C (mg/dL)]) (20).

### Follow-up and assessment of outcomes

The primary outcome was the first occurrence of stroke (ischemic or hemorrhagic), either nonfatal or fatal. Secondary outcomes included the first ischemic stroke and the first hemorrhagic stroke. The outcomes of events since participants enrolled in the study at baseline were determined through medical records, contact with local disease and death registries, or access to the database of basic medical insurance. These data sources are linked using an individual national identification number assigned to each Chinese person for life. This number is replaced by a series number when provided for personal data analysis to anonymize the individual participant’s data. Patients were followed from the date of enrollment to the end of the observation period, defined as the date of the last follow-up visit, the date of the first appearance of any study outcome, the date of death, or the end of the study period (December 31, 2021).

### Statistical analysis

We compared the METS-IR quartile characteristics of the participants. The cumulative incidence of total stroke and its subtypes was estimated using the Kaplan-Meier method. Covariates with variance inflation factors (VIF) ≥ 5 were omitted to avoid multicollinearity (**Table S3**). Hazard ratios (HR) and confidence intervals (CI) were derived from the Cox regression models. Restricted cubic spline (RCS) curves were created at the 10th, 50th, and 90th percentiles using three default knots. In addition, we performed subgroup analyses stratified by age, sex, eGFR, Hcy, hyperlipidemia, diabetes, and CCI. Interactions between METS-IR and each of these variables were tested. We conducted several sensitivity analyses to test the robustness of our findings. First, we excluded events occurring in the first two years of follow-up to minimize potential reverse causality. In the second sensitivity analysis, we additionally excluded any participants older than 80 years. Third, the same analyses were repeated after excluding participants under treatment with glucose-lowering or lipid-lowering medications. Fourth, competing risk analyses were performed using the Fine and Gray method, and non-stroke deaths were treated as competing risk events. Fifth, a sensitivity analysis without adjustment for diabetes and hyperlipidemia was used to exclude potential bias. Finally, we also performed a sensitivity analysis using an E-value approach. Details of the statistical analysis are provided in the **Supplementary Material**. All analyses were done with R software version 4.1.1 at a two-tailed alpha level of 0.05.

## Results

### Characteristics of the study population

The METS-IR was normally distributed in the population (**Figure S1**). Participants were divided into four groups based on METS-IR quartiles at baseline (**Table 1**). Among the 14032 participants eligible for analysis, individuals with higher METS-IR levels were younger, more likely to be current smokers and drinkers, had a higher BMI, and had higher rates of hyperlipidemia, CHD, and diabetes. Furthermore, participants with higher METS-IR levels used glucose-lowering medications and statins more frequently during treatment (**Table S4**).

**Table 1:** Baseline characteristics of the study population according to quartiles of METS-IR.

Characteristics	METS-IR quartiles				*P*-value
	Q1 (<37.32)	Q2 (37.32-42.47)	Q3 (42.48-48.22)	Q4 (>48.22)	
Participants, n	3507	3507	3508	3510	
Age, year	52.36 ± 12.20	51.57 ± 12.07	52.31 ± 11.74	51.90 ± 11.93	0.018
Men, n (%)	1904 (54.29%)	1879 (53.58%)	1931 (55.05%)	1915 (54.56%)	<0.001
Body mass index, kg/m^2^	22.61 ± 1.61	24.74 ± 1.45	26.61 ± 1.86	29.59 ± 2.89	<0.001
Heart rate, bpm	80.41 ± 10.85	80.67 ± 10.20	81.22 ± 10.18	82.82 ± 10.78	<0.001
SBP, mmHg	143.97 ± 20.51	145.43 ± 20.57	145.92 ± 20.46	148.07 ± 20.96	<0.001
DBP, mmHg	87.53 ± 14.35	89.22 ± 14.28	90.34 ± 14.17	92.61 ± 14.67	<0.001
Current smoking, n (%)	615 (17.54%)	1082 (30.85%)	1300 (37.06%)	1526 (43.48%)	<0.001
Current drinking, n (%)	572 (16.31%)	1013 (28.89%)	1234 (35.18%)	1390 (39.60%)	<0.001
Laboratory parameters					
UA, mmol/L	293.85 ± 81.26	330.34 ± 84.67	355.05 ± 91.51	377.28 ± 99.33	<0.001
eGFR, mL/min/1.73 m^2^	97.14 ± 18.64	96.47 ± 17.55	96.68 ± 17.51	97.35 ± 18.55	0.153
Cys C, mg/L	0.90 ± 0.34	0.92 ± 0.33	0.93 ± 0.34	0.95 ± 0.31	<0.001
TC, mmol/L	4.45 ± 0.95	4.42 ± 0.94	4.48 ± 1.00	4.53 ± 1.04	<0.001
TG, mmol/L	1.19 ± 0.52	1.60 ± 0.83	1.96 ± 1.04	2.74 ± 2.02	<0.001
HDL-C, mmol/L	1.31 ± 0.30	1.09 ± 0.22	0.99 ± 0.20	0.88 ± 0.18	<0.001
LDL-C, mmol/L	2.65 ± 0.82	2.78 ± 0.81	2.75 ± 0.85	2.71 ± 0.83	<0.001
HbA1c, %	6.01 ± 1.22	6.17 ± 1.25	6.18 ± 1.24	6.22 ± 1.26	<0.001
FPG, mmol/L	4.68 ± 0.94	5.03 ± 1.51	5.36 ± 2.02	5.84 ± 2.36	<0.001
Hcy, mmol/L	14.40 ± 6.92	14.58 ± 6.79	14.96 ± 7.04	15.08 ± 7.33	<0.001
hsCRP, mg/L	3.14 (1.23-7.45)	3.02 (1.15-7.17)	3.15 (1.15-7.65)	3.27 (1.17-7.80)	0.319
Medical histories, n (%)					
Hyperlipidemia	1982 (56.52%)	2010 (57.31%)	2072 (59.06%)	2140 (60.97%)	<0.001
Coronary heart disease	489 (13.94%)	435 (12.40%)	541 (15.42%)	708 (20.17%)	<0.001
Diabetes	965 (27.52%)	923 (26.32%)	1001 (28.53%)	1127 (32.11%)	<0.001
Charlson comorbidity index					0.005
0	1622 (46.25%)	1637 (46.68%)	1602 (45.67%)	1532 (43.65%)	
1	944 (26.92%)	1029 (29.34%)	1037 (29.56%)	1051 (29.94%)	
2 or more	941 (26.83%)	841 (23.98%)	869 (24.77%)	927 (26.41%)	
Follow-up duration, years	4.80 (1.80-7.50)	4.70 (1.70-7.60)	4.80 (1.80-7.50)	5.00 (1.80-7.60)	0.332

Data are mean (standard deviation), n (%), or median (interquartile range).

METS-IR, metabolic score for insulin resistance; SBP, systolic blood pressure; DBP, diastolic blood pressure; eGFR, estimated glomerular fltration rate; TC, total cholesterol; TG, triglyceride; HDL-C, high-density lipoprotein cholesterol; LDL-C, low-density lipoprotein cholesterol; HbA1c, hemoglobin A1c; FPG, fasting plasma glucose; Hcy, homocysteine; UA, uric acid; hsCRP, high-sensitivity C-reactive protein; Cys C, cystatin C.

### Association of METS-IR with total stroke and its subtypes

**Figure 2:**
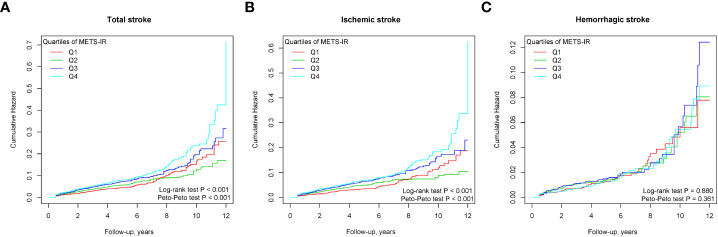
Cumulative incidence of outcomes stratified by the quartile of the METS-IR. **(A)** total stroke; **(B)** ischemic stroke; **(C)** hemorrhagic stroke.

**Figure 3:**
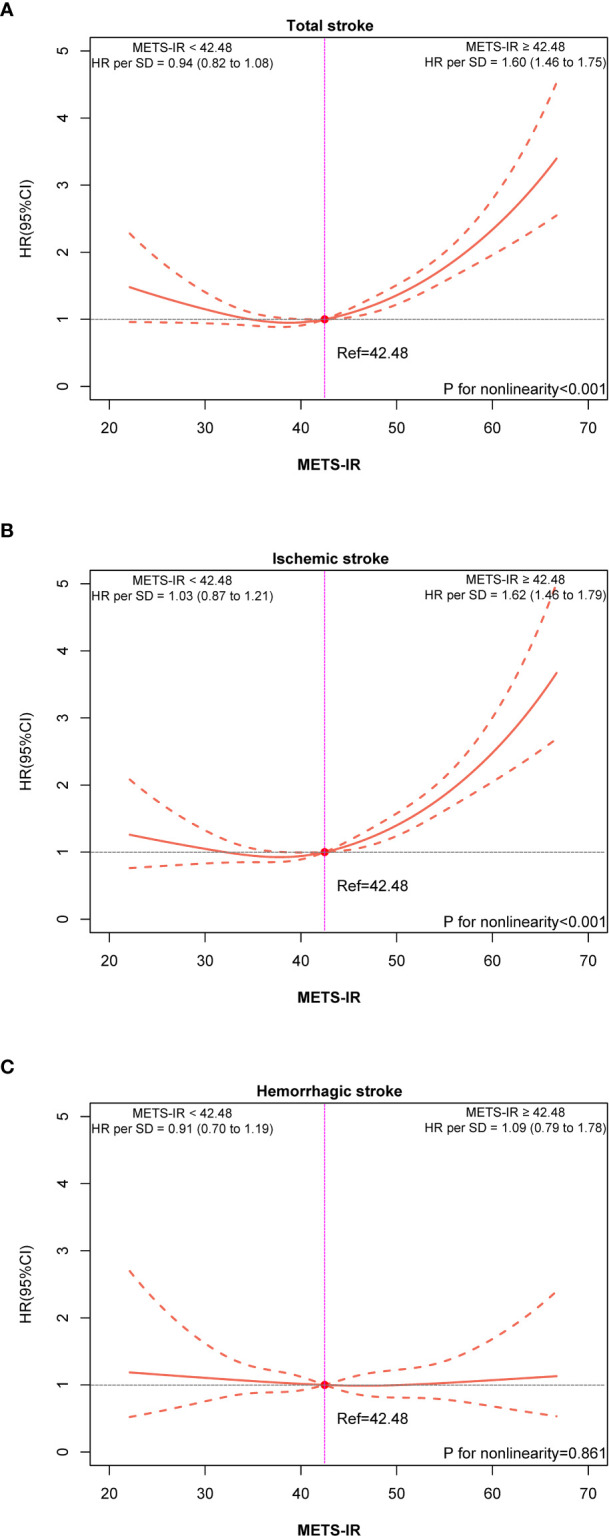
Dose-response associations of METS-IR with total stroke **(A)**, ischemic stroke **(B)**, and hemorrhagic stroke **(C)**.

During a median follow-up of 4.80 years (interquartile range, 1.80-7.60), among the eligible participants, 1067 patients had a total stroke, including 842 incident ischemic strokes and 225 incident hemorrhagic strokes. The incidence rates of total stroke, ischemic stroke, and hemorrhagic stroke were 15.45 (95% CI: 14.54–16.41), 12.47 (95% CI: 11.89–13.07), and 3.98 (95% CI: 3.66-4.33) per 1000 person-years, respectively. The Kaplan-Meier curve showed that participants in the Q4 group had a higher risk of total stroke and ischemic stroke instead of hemorrhagic stroke than those in other groups (log-rank test, P < 0.001, **Figures 2A, B**; P = 0.880, **Figure 2C**) (Peto-Peto test, P < 0.001, **Figures 2A, B**; P = 0.361, **Figure 2C**). The cumulative incidence of total stroke increased with increasing METS-IR (**Figure 2A**). This trend remained significant even after adjusting for potential confounders in model 3 (P trend < 0.001). Compared with the Q1 group, the HRs were 0.97 (95% CI, 0.80-1.18), 1.34 (95% CI, 1.12-1.61), and 1.80 (95% CI, 1.50-2.17) for the Q2, Q3, and Q4 groups, respectively (**Table 2**). It appeared that the risk of total stroke was higher per 1 SD increase of METS-IR (HR, 1.33; 95% CI, 1.25-1.42; **Table 2**). Similar results were seen in ischemic stroke, but the risk of hemorrhagic stroke was not significantly increased (**Table 2**). To visualize the relationship between the METS-IR and total stroke and its subtypes, we fitted 3 RCS curves (**Figure 3**). The multivariable-adjusted spline regression model showed a nearly J-shaped dose-response relationship between the METS-IR and the risk of total stroke (P for nonlinearity < 0.001). A similar association has been found in ischemic stroke. In contrast, the association between METS-IR and incident hemorrhagic stroke risk was nonlinear (P for nonlinearity = 0.861). As METS-IR increased beyond 42.48, the HRs for both total stroke (HR per SD 1.60, 95% CI 1.46-1.75) and ischemic stroke (HR per SD 1.62, 95% CI 1.46-1.79) increased significantly.

**Table 2:** Associations between METS-IR and clinical outcomes.

Exposure	Unadjusted	Model 1	Model 2	Model 3
	HR (95% CI)	HR (95% CI)	HR (95% CI)	HR (95% CI)
**Total stroke**				
Per SD increment	1.26 (1.19, 1.33)	1.26 (1.19, 1.34)	1.29 (1.21, 1.37)	1.33 (1.25, 1.42)
Categories				
Q1-Q2	Reference	Reference	Reference	Reference
Q3-Q4	1.49 (1.32, 1.68)	1.48 (1.31, 1.67)	1.52 (1.34, 1.72)	1.57 (1.38, 1.79)
Quartiles				
Q1	Reference	Reference	Reference	Reference
Q2	0.95 (0.79, 1.15)	0.97 (0.80, 1.18)	0.96 (0.80, 1.17)	0.97 (0.80, 1.18)
Q3	1.30 (1.09, 1.55)	1.29 (1.08, 1.54)	1.30 (1.09, 1.55)	1.34 (1.12, 1.61)
Q4	1.60 (1.35, 1.90)	1.63 (1.38, 1.93)	1.71 (1.43, 2.03)	1.80 (1.50, 2.17)
P for trend	<0.001	<0.001	<0.001	<0.001
**Ischemic stroke**				
Per SD increment	1.32 (1.23, 1.41)	1.32 (1.24, 1.41)	1.35 (1.26, 1.44)	1.39 (1.29, 1.49)
Categories				
Q1-Q2	Reference	Reference	Reference	Reference
Q3-Q4	1.61 (1.40, 1.85)	1.60 (1.39, 1.84)	1.63 (1.41, 1.87)	1.68 (1.45, 1.95)
Quartiles				
Q1	Reference	Reference	Reference	Reference
Q2	1.01 (0.81, 1.25)	1.03 (0.83, 1.28)	1.03 (0.83, 1.28)	1.04 (0.84, 1.30)
Q3	1.47 (1.21, 1.79)	1.46 (1.20, 1.78)	1.47 (1.20, 1.80)	1.52 (1.24, 1.87)
Q4	1.75 (1.45, 2.12)	1.79 (1.48, 2.17)	1.85 (1.52, 2.26)	1.96 (1.60, 2.42)
P for trend	<0.001	<0.001	<0.001	<0.001
**Hemorrhagic stroke**				
Per SD increment	0.98 (0.87, 1.11)	0.98 (0.87, 1.11)	1.02 (0.90, 1.15)	1.01 (0.89, 1.15)
Categories				
Q1-Q2	Reference	Reference	Reference	Reference
Q3-Q4	1.06 (0.83, 1.34)	1.05 (0.83, 1.33)	1.12 (0.87, 1.42)	1.12 (0.87, 1.44)
Quartiles				
Q1	Reference	Reference	Reference	Reference
Q2	1.02 (0.73, 1.44)	1.02 (0.73, 1.44)	1.01 (0.71, 1.42)	0.96 (0.68, 1.35)
Q3	1.13 (0.81, 1.57)	1.12 (0.80, 1.56)	1.13 (0.81, 1.59)	1.12 (0.79, 1.58)
Q4	1.01 (0.72, 1.42)	1.01 (0.72, 1.42)	1.10 (0.78, 1.57)	1.07 (0.74, 1.55)
P for trend	0.817	0.835	0.462	0.530

Model 1, adjusted for age; sex, Model 2, adjusted for heart rate, SBP, DBP, current smoker, current drinker, hyperlipidemia, Charlson comorbidity index, diabetes, and coronary heart disease based on model 1, Model 3; included variables in model 2 and further adjusted for uric acid, eGFR, cystatin C, TC, TG, LDL-C, HbA1c, FPG, Hcy, hsCRP, use of statins, use of aspirins, use of insulins, use of oral antidiabetic drugs, and antihypertensive drugs.

SD, standard deviation; HR, hazard ratio; CI, confidence interval. Other abbreviations as presented in **Table 1**.

### Stratified analyses

**Figure 4:**
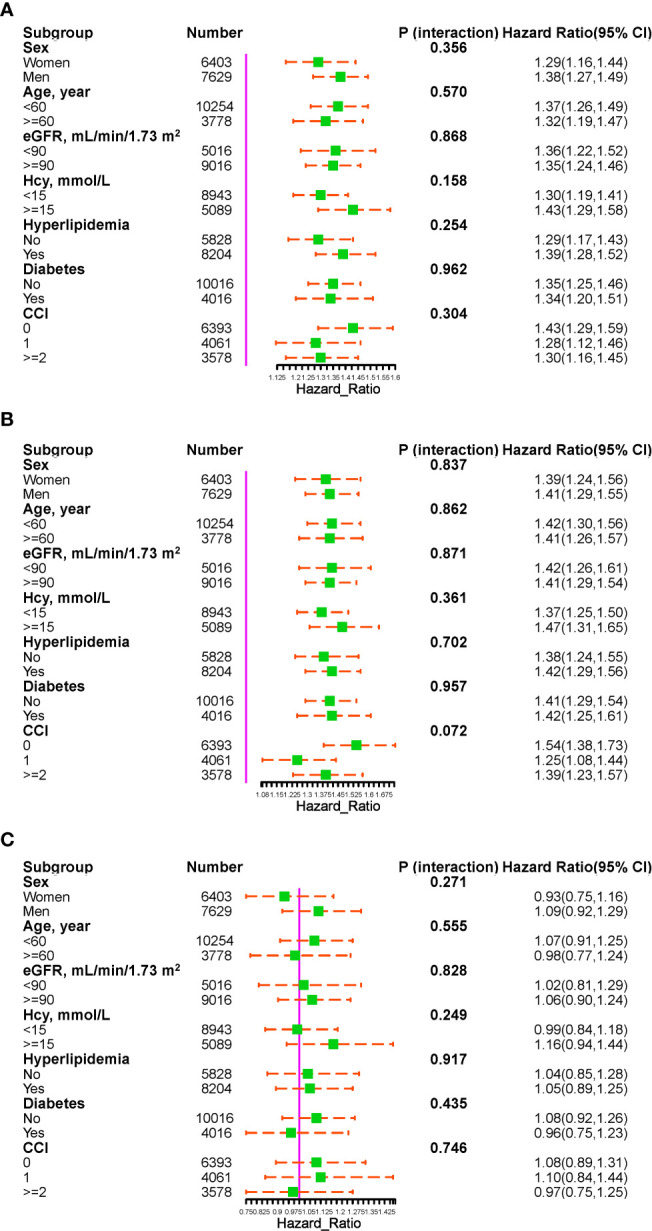
The association of METS-IR (per SD increment) with the risk of total stroke **(A)**, ischemic stroke **(B)**, and hemorrhagic stroke **(C)** in various subgroups.

Stratified analysis was conducted to evaluate the association of METS-IR (per SD increase) with the risk of total stroke in each subgroup (**Figure 4**). None of the factors significantly altered the association between METS-IR and the risk of total stroke (all P for interactions > 0.05). A stratified analysis of the association between METS-IR (per SD increase) and the risk of ischemic stroke found similar trends (**Figure 4B**).

### Sensitivity analysis

We performed sensitivity analyses to confirm the effect of METS-IR on total stroke and its subtypes in patients with hypertension. **Tables S5**–**S10** present results from our sensitivity analyses. In the sensitivity analyses, the associations of METS-IR with the risk of total stroke and its subtypes did not change significantly after excluding participants who had an outcome event during the first two years of follow-up (**Table S5**), excluding participants aged 80 and older (**Table S6**), excluding participants under treatment with glucose-lowering medications (**Table S7**), or excluding participants receiving lipid-lowering therapy (**Table S8**). In analyses with non-stroke death as a competing risk, there was no significant change in the primary outcome (**Table S9**). The primary outcome was not changed using multiple regression analysis without adjustment for diabetes and hyperlipidemia (**Table S10**). The E-values demonstrated that the observed correlations were at least moderately robust to potential unmeasured confounding (**Table S11**).

### Incremental predictive value of METS-IR

As demonstrated in **Table 3**, METS-IR did produce a significant improvement in the C statistic when added to the basic model (from 0.637 to 0.664, P < 0.001). Notably, the addition of METS-IR to the basic model resulted in a significant improvement in predicting incident total stroke, with increments in continuous NRI (0.114, P < 0.001) and IDI (0.007, P = 0.007). In ischemic stroke, similar findings were observed.

**Table 3:** Incremental predictive value of METS-IR.

	C statistic		NRI (continuous)		IDI	
	Estimate (95% CI)	*P*-value	Estimate (95% CI)	*P*-value	Estimate (95% CI)	*P*-value
**Total stroke**						
Basic model	0.637 (0.621–0.654)		Reference		Reference	
Basic model + METS-IR	0.664 (0.648–0.681)	<0.001	0.114 (0.028–0.157)	<0.001	0.007 (0.001–0.010)	0.007
**Ischemic stroke**						
Basic model	0.664 (0.646–0.683)		Reference		Reference	
Basic model + METS-IR	0.686 (0.669–0.704)	<0.001	0.132 (0.030–0.167)	<0.001	0.005 (0.001–0.008)	0.033
**Hemorrhagic stroke**						
Basic model	0.596 (0.564–0.628)		Reference		Reference	
Basic model + METS-IR	0.625 (0.593–0.657)	0.066	0.055 (-0.110–0.128)	0.698	0.001 (-0.004–0.003)	0.944

The basic model included age, sex, heart rate, SBP, DBP, current smoker, current drinker, hyperlipidemia, Charlson comorbidity index, diabetes, coronary heart disease, uric acid, eGFR, cystatin C, TC, TG, LDL-C, HbA1c, FPG, Hcy, and hsCRP.

IDI, integrated discrimination improvement; NRI, net reclassification index. Other abbreviations as presented in **Table 1**.

## Discussion

In this large retrospective cohort study, the risk of stroke and its subtypes based on the METS-IR, a novel surrogate marker of IR, was assessed. We consistently found that higher levels of METS-IR at baseline were associated with an increased risk of future stroke and ischemic stroke, even after adjusting for confounders. However, there was no significant correlation between baseline METS-IR and hemorrhagic stroke. Additionally, we observed a nearly J-shaped association between levels of METS-IR and the risk of stroke and ischemic stroke.

IR is an essential indicator of metabolic abnormalities (24). In the long term, IR can lead not only naturally to pathophysiological disorders such as abnormal glucolipid metabolism, elevated blood pressure, hyperuricemia, raised signatures of inflammation, and thrombotic states, but also indirectly to diseases associated with metabolic disorders (25–27). Recently, a new non-insulin metabolic score based on conventional clinical indicators such as FPG, TG, HDL-C, and BMI, namely METS-IR, was developed and has been shown to have a high accuracy similar to that of EHC (17, 20). Bello-Chavolla et al. analyzed the advantages of METS-IR versus markers such as EHC and TyG in the diagnosis of impaired insulin sensitivity and demonstrated that METS-IR was significantly better than the other markers (17, 20). Research to indicate that METS-IR may be used to screen for early insulin sensitivity and metabolism-related illnesses (17). In a large cohort study, Lee et al. demonstrated that METS-IR was superior to HOMA-IR in predicting the incidence of NAFLD and that METS-IR may be a more accurate index of IR than HOMA-IR (28). In another large epidemiological study, Liu et al. identified elevated METS-IR with a concomitant increased risk of hypertension (29). In a community-based population without cardiovascular disease, a J-shaped association was found between METS-IR and subclinical myocardial injury (16). The results of Wu et al. suggest that METS-IR is a significant predictor of the presence and severity of CHD and may serve as a quality indicator for the prevention and management of CHD (22). A cohort study in Korea also demonstrated that elevated METS-IR predicted the future risk of ischemic heart disease in a community-based population without diabetes and served as a useful predictive marker for ischemic heart disease (30). In addition, studies revealed that METS-IR is also strongly associated with many risk factors for stroke, such as hyperuricemia, atherosclerosis, and early renal insufficiency (18, 31–34). In summary, METS-IR may be an economical and convenient index for IR screening. Our findings suggest that elevated METS-IR may be useful in identifying people at high risk for developing stroke. In terms of clinical applications, contemporary electronic medical records have the potential to automatically calculate METS-IR in order to better stratify individuals by risk based on METS-IR. A high METS-IR can also alert people to establish early lifestyle changes that can reduce disease progression or morbidity.

Mechanisms linking METS-IR and stroke and ischemic stroke remain incompletely understood. There are several potential interpretations for this observation. First, IR enhances the atherosclerotic process. IR enhances the pathophysiological processes of vascular endothelial cells, smooth muscle cells, and macrophages *via* inflammation, promoting the formation of atherosclerosis-associated foam cells and vulnerable plaques. In addition, IR may have atherogenic effects through impaired fibrinolysis and dyslipidemia (35–38). Second, it has been shown that IR plays an instrumental function in platelet adhesion, activation, and aggregation (39–41). IR may increase platelet count and volume and promote platelet activation. Moreover, IR is tightly linked to vascular endothelial dysfunction, which further promotes platelet adhesion and aggregation (41–43). All of the above are intimately correlated with cerebral vascular stenosis or occlusion and are involved in ischemic stroke events. Third, IR predisposes to hemodynamic disturbances. Previous studies have found significantly reduced cerebrovascular reserve in insulin-resistant patients (44–46). Finally, IR may accelerate the progression of atherosclerosis by altering risk factors and disrupting brain metabolism through oxidative stress and inflammatory mechanisms (47–49). Further examinations are warranted to clarify the precise role of METS-IR in stroke and ischemic stroke in the future. Nevertheless, no association between METS-IR and hemorrhagic stroke was observed in this study. Although hypertension is an independent risk factor for hemorrhagic stroke, it has been proposed that lipid metabolism disorders can produce a protective effect against cerebrovascular hemorrhage (50). Thus, the combination of two opposing effects may have contributed to the statistically nonsignificant association between METS-IR and hemorrhagic stroke in hypertensive patients.

This study has several strengths that distinguish it from previous studies. First, to our knowledge, this is the first large cohort study to assess the association between METS-IR and the risk of stroke and its subtypes in patients with hypertension. Second, this study reports the results derived from real-world clinical practice. Our findings are more likely to reflect real-world conditions. Several potential limitations are also noteworthy. First, the observational, retrospective study design limits inferences of causality. Second, the participants in this study were mainly Chinese hypertensive patients, so it is uncertain whether the obtained results would be generalizable to other populations. Although we controlled for confounders, we cannot rule out the possibility that unmeasured (e.g., genetic susceptibility and environmental exposure) or poorly measured confounders could explain our observations. Thus, further prospective studies are needed to confirm these findings. Finally, this study does not use repeated measurements of METS-IR. Longitudinal studies using repeated measurements of METS-IR are necessary to investigate more accurate associations between METS-IR and outcomes than a single measurement.

## Conclusion

In summary, a relationship between METS-IR and the risk of stroke and ischemic stroke was observed in patients with hypertension. It will require further studies to clarify this potential mechanism.

## Data availability statement

The original contributions presented in the study are included in the article/**Supplementary Material**. Further inquiries can be directed to the corresponding author.

## Ethics statement

The studies involving human participants were reviewed and approved by People’s Hospital of Xinjiang Uygur Autonomous Region. Written informed consent for participation was not required for this study in accordance with the national legislation and the institutional requirements.

## Author contributions

XC and JHu analyzed the data and wrote the manuscript. XC, QZ, MW, and YD helped with copyediting. XC and NL audited the data. SL, JHo, and XC conducted research. NL had primary responsibility for the final content of the manuscript. All authors read and approved the final manuscript.

## Funding

This research was supported by the Chinese Academy of Medical Sciences (2020-RW330-002).

## Acknowledgments

We are grateful to the Medical Insurance Administration Bureau of Xinjiang Uygur Autonomous Region for providing us access to the database of basic medical insurance.

## Conflict of interest

The authors declare that the research was conducted in the absence of any commercial or financial relationships that could be construed as a potential conflict of interest.

## Publisher’s note

All claims expressed in this article are solely those of the authors and do not necessarily represent those of their affiliated organizations, or those of the publisher, the editors and the reviewers. Any product that may be evaluated in this article, or claim that may be made by its manufacturer, is not guaranteed or endorsed by the publisher.
