# Supplementary material for: Relationship of the metabolic score for insulin resistance and the risk of stroke in patients with hypertension: A cohort study
Source: Front Endocrinol (Lausanne). 2022 Dec 5;13:1049211. doi: 10.3389/fendo.2022.1049211 (PMC9760826; doi:10.3389/fendo.2022.1049211)
Supplement: Supplementary file 1 [file DataSheet_1.docx]

Supplementary Material

# Supplemental material and methods

**1.1 Details of the statistical analyses**.

Baseline characteristics were analyzed using descriptive statistics. Missing values of covariates were imputed using multiple imputations by chained equations. A normality test (Kolmogorov–Smirnov test) was primarily used for measurement data. Categorical variables were described as frequency and percentage. Continuous variables were described as mean (± standard deviation [SD]) for normally distributed data and as geometric mean and 95% confidential interval (CI) for data not normally distributed. We compared the baseline METS-IR quartile characteristics of participants. Continuous variables were compared using one-way ANOVA, while categorical variables were compared using the chi-square test.

Kaplan-Meier method was used to compute cumulative incidence of stroke and subgroup of stroke. Differences between Kaplan-Meier curves were compared using the logrank test and the Peto-Peto-Prentice test. To avoid the result deviation caused by the collinearity of variables, the variance inflation factor (VIF) was calculated. Variables with VIF ≥ 5 were excluded to avoid multi-collinearity. Schoenfeld residuals were generated to confirm the risk proportionality assumptions. The proportional hazards assumption was met for all Cox and competing risk models. Multivariate Cox proportional hazard models were used to obtain the hazard ratios (HRs) to assess the risks of total stroke, ischemic stroke, and hemorrhagic stroke. In the multivariate models, we adjusted for age and sex in model 1. In model 2, we further adjusted for heart rate, SBP, DBP, smoking status, drinking status, hyperlipidemia, CCI, diabetes, and CHD. In model 3, we further adjusted for UA, eGFR, Cys C, TC, TG, LDL-C, HbA1c, FPG, Hcy, and hsCRP. The linear trend was tested by assigning a median value for each group as a continuous variable.

To capture the dose-response relationship between METS-IR and the risk of stroke, restricted cubic spline models were used, with three knots at the 10th, 50th, and 90th percentiles of METS-IR. If non-linear relationships were found, two-piecewise linear regression models would be used to illustrate how the relationships differ by threshold point. The threshold value was estimated by trying all possible values and choosing the threshold value with the highest likelihood. A logarithmic likelihood ratio test was employed to compare the one-line linear regression model with a two-piecewise linear model.

Stratified analyses were also conducted by age (< 60 or ≥ 60 years), sex (men or women), BMI (< 24 or ≥24, <28 or ≥28 kg/m^2^), eGFR (< 90 or ≥ 90 mL/min/1.73 m^2^), Hcy (< 15 or ≥ 15 mmol/L), hyperlipidemia (yes or no), diabetes (yes or no), CCI (0 or 1 or ≥2). The potential effects of such modifications were evaluated by modeling the cross-product of the stratification variable with the METS-IR.

We performed several sensitivity analyses to test the robustness of our findings. First, we excluded events occurring in the first 2 years of follow-up to minimize potential reverse causality. Second, patients with diabetes were excluded to control against any potential biases induced by baseline conditions. Third, we implemented a competing risk model that considered non-stroke deaths as competing risk events. In the fourth sensitivity analysis, we additionally excluded any participants older than 80 years. Fifth, we also performed a sensitivity analysis using an E-value approach. Finally, we further adjusted for medication use during the treatment period.

Furthermore, we used the C statistic, net reclassification index (NRI), and integrated discrimination improvement (IDI) to assess the incremental predictive value of METS-IR changes beyond the basic model. The basic model included age, sex, heart rate, SBP, DBP, current smoker, current drinker, hyperlipidemia, Charlson comorbidity index, diabetes, coronary heart disease, uric acid, eGFR, cystatin C, TC, TG, LDL-C, HbA1c, FPG, Hcy, and hsCRP.

P values were 2-sided, and statistical significance was set at P < .05. All analyses were performed using R software version 4.1.1.

# Supplementary Figures and Tables

## Supplementary Tables

**Table S1**. Characteristics of the included and excluded population in the current study *

|  | **Included** | **Excluded due to missing information** | **Excluded due to history of stroke or other diseases** |
| --- | --- | --- | --- |
| **Characteristics** | **(n=14032)** | **(n=**1531**)** | **(n=1215)** |
| Age, year | 52.03 ± 11.99 | 51.49 ± 17.56 | 53.22 ± 18.89 |
| Men, % | 54 | 52 | 56 |
| Heart rate, bpm | 81.28 ± 10.55 | 80.82 ± 16.49 | 81.89 ± 18.50 |
| DBP, mmHg | 89.92 ± 14.49 | 88.18 ± 18.89 | 91.24 ± 19.76 |
| SBP, mmHg | 145.85 ± 20.67 | 144.87 ± 25.09 | 145.67 ± 27.65 |
| Body mass index, kg/m^2^ | 25.89 ± 3.27 | 25.79 ± 8.54 | 25.23 ± 9.34 |
| Current smoking, % | 32 | 34 | 30 |
| Current drinking, % | 30 | 32 | 28 |
| Uric acid, mmol/L | 339.14 ± 94.67 | 342.51 ± 103.63 | 336.65 ± 106.41 |
| eGFR, mL/min/1.73 m2 | 96.91 ± 18.07 | 94.82 ± 26.57 | 98.56 ± 27.58 |
| Cystatin C, mg/L | 0.92 ± 0.33 | 0.91 ± 0.41 | 0.95 ± 0.43 |
| TC, mmol/L | 4.47 ± 0.98 | 4.45 ± 1.24 | 4.49 ± 1.61 |
| TG, mmol/L | 1.87 ± 1.36 | 1.89 ± 1.89 | 1.84 ± 2.14 |
| HDL-C, mmol/L | 1.07 ± 0.28 | 1.09 ± 0.56 | 1.02 ± 0.69 |
| LDL-C, mmol/L | 2.72 ± 0.83 | 2.71 ± 1.22 | 2.68 ± 1.31 |
| HbA1c, % | 6.14 ± 1.24 | 6.18 ± 1.71 | 6.11 ± 1.83 |
| FPG, mmol/L | 5.23 ± 1.84 | 5.24 ± 2.29 | 5.22 ± 2.67 |
| Hcy, mmol/L | 14.76 ± 7.03 | 14.95 ± 7.67 | 14.27 ± 7.81 |
| hsCRP, mg/L | 5.35 ± 6.61 | 5.27 ± 7.26 | 5.36 ± 7.12 |

Abbreviations as presented in Table 1.

**Table S2**. List of medications included in the study.

| Drug class | Drug name |
| --- | --- |
| Aspirin | Aspirin |
| Beta-blocker | Atenolol, bisoprolol, carvedilol, metoprolol, propranolol |
| Angiotensin-converting enzyme inhibitors or angiotensin receptor blockers | Azilsartan, candestartan, captopril, enalapril, fosinopril, irbesartan, losartan, olmesartan, ramipril telmisartan, valsartan |
| Calcium channel blockers | Amlodipine, diltiazem, felodipine, lercanidipine, nifedipine,  verapamil |
| Diuretics | Acetazolamide, amiloride, benzyl hydrochlorothiazide, bumetanide, furosemide, hydrochlorothiazide, indapamide, spironolactone |
| Statin | Atorvastatin, fluvastatin, pitavastatin, rosuvastatin, simvastatin |
| Oral antidiabetic agents | Metformin, glipizide, gliclazide, glimepiride, glyburide, alogliptin, linagliptin, sitagliptin, vidagliptin, saxagliptin, acarbose, nateglinide, meglitinide, repaglinde, pioglitzone,dulaglutide, exenatide, liraglutide |
| Insulin | Rapid, short, intermediate and long-acting insulins |

**Table S3**. Collinearity diagnostics steps.

|  | Step 1 | Step 2 |
| --- | --- | --- |
| METS-IR | 33 | 2 |
| Gender | 1.1 | 1.1 |
| Age | 1.2 | 1.2 |
| Hyperlipidemia | 1.1 | 1.1 |
| Coronary heart disease | 1.2 | 1.2 |
| Diabetes | 1.7 | 1.7 |
| Charlson Comorbidity Index | 1.9 | 1.9 |
| Current smoking | 1.7 | 1.7 |
| Current drinking | 1.7 | 1.7 |
| Heart rate | 1 | 1 |
| DBP | 1.9 | 1.9 |
| SBP | 1.9 | 1.9 |
| Body mass index | 18.5 | NA |
| Uric acid | 1.4 | 1.4 |
| eGFR | 1 | 1 |
| Cyctatin C | 1.1 | 1.1 |
| TC | 1.7 | 1.7 |
| TG | 3.4 | 1.6 |
| HDL-C | 5.3 | NA |
| LDL-C | 1.3 | 1.3 |
| HbA1c | 1.1 | 1.1 |
| FPG | 1.7 | 1.1 |
| Hcy | 1.1 | 1.1 |
| hsCRP | 1.4 | 1.4 |

VIF = 1/(1-R^2^). We employed multiple linear regression, starting with including all the covariates and incrementally removing the variables with highest variance inflation factor until achieving the threshold value (all covariates with a variance inflation factor below 5).

Abbreviations: VIF, variance inflation factors. Other abbreviations as presented in Table 1.

**Table S4.** Concomitant medication usage during the treatment period according to METS-IR quartiles *.

| Medication use, n (%) | METS-IR quartiles | | | | P-value |
| --- | --- | --- | --- | --- | --- |
|  | Q1 | Q2 | Q3 | Q4 |  |
| Statin | 900 (25.66%) | 1573 (44.85%) | 1585 (45.18%) | 1882 (53.62%) | <0.001 |
| Aspirin | 2323 (66.24%) | 2286 (65.18%) | 2296 (65.45%) | 2336 (66.55%) | 0.583 |
| Beta blockers | 1349 (38.47%) | 1365 (38.92%) | 1400 (39.91%) | 1419 (40.43%) | 0.315 |
| Calcium channel blockers | 2792 (79.61%) | 2769 (78.96%) | 2767 (78.88%) | 2822 (80.40%) | 0.359 |
| ACEI/ARB | 2600 (74.14%) | 2593 (73.94%) | 2593 (73.92%) | 2566 (73.11%) | 0.768 |
| Diuretics | 806 (22.98%) | 769 (21.93%) | 789 (22.49%) | 828 (23.59%) | 0.392 |
| Oral antidiabetic agents | 586 (16.71%) | 608 (17.34%) | 641 (18.27%) | 819 (23.33%) | <0.001 |
| Insulin | 328 (9.35%) | 306 (8.73%) | 357 (10.18%) | 564 (16.07%) | <0.001 |

*Regular concomitant medication was defined as 180 or more cumulative days of taking the drug of interest.

Abbreviations as presented in Table S2.

**Table S5**. Sensitivity analysis of excluding outcome events within the first two years of follow-up.

| Exposure | Unadjusted | Model 1 | Model 2 | Model 3 |
| --- | --- | --- | --- | --- |
|  | HR (95% CI) | HR (95% CI) | HR (95% CI) | HR (95% CI) |
| **Total stroke** |  |  |  |  |
| Per SD increment | 1.21 (1.13, 1.30) | 1.22 (1.13, 1.31) | 1.24 (1.15, 1.33) | 1.27 (1.17, 1.37) |
| Quartiles |  |  |  |  |
| Q1 | Reference | Reference | Reference | Reference |
| Q2 | 0.79 (0.63, 1.00) | 0.81 (0.64, 1.02) | 0.80 (0.64, 1.01) | 0.79 (0.63, 1.00) |
| Q3 | 1.12 (0.91, 1.38) | 1.11 (0.90, 1.37) | 1.11 (0.89, 1.37) | 1.13 (0.91, 1.40) |
| Q4 | 1.43 (1.17, 1.74) | 1.46 (1.20, 1.78) | 1.50 (1.22, 1.85) | 1.57 (1.27, 1.96) |
| P for trend | <0.001 | <0.001 | <0.001 | <0.001 |
| **Ischemic stroke** |  |  |  |  |
| Per SD increment | 1.26 (1.16, 1.37) | 1.27 (1.17, 1.37) | 1.28 (1.17, 1.39) | 1.31 (1.20, 1.44) |
| Quartiles |  |  |  |  |
| Q1 | Reference | Reference | Reference | Reference |
| Q2 | 0.80 (0.61, 1.05) | 0.83 (0.63, 1.08) | 0.83 (0.63, 1.08) | 0.83 (0.63, 1.09) |
| Q3 | 1.29 (1.02, 1.64) | 1.28 (1.01, 1.62) | 1.27 (1.00, 1.62) | 1.30 (1.01, 1.66) |
| Q4 | 1.53 (1.22, 1.93) | 1.58 (1.25, 1.98) | 1.59 (1.26, 2.02) | 1.68 (1.31, 2.15) |
| P for trend | <0.001 | <0.001 | <0.001 | <0.001 |
| **Hemorrhagic stroke** |  |  |  |  |
| Per SD increment | 1.00 (0.87, 1.15) | 1.00 (0.86, 1.14) | 1.02 (0.88, 1.18) | 1.02 (0.87, 1.19) |
| Quartiles |  |  |  |  |
| Q1 | Reference | Reference | Reference | Reference |
| Q2 | 0.89 (0.60, 1.32) | 0.89 (0.59, 1.32) | 0.87 (0.58, 1.29) | 0.81 (0.54, 1.21) |
| Q3 | 0.95 (0.64, 1.40) | 0.94 (0.64, 1.39) | 0.93 (0.63, 1.39) | 0.92 (0.61, 1.39) |
| Q4 | 0.99 (0.67, 1.45) | 0.99 (0.67, 1.45) | 1.05 (0.70, 1.57) | 1.01 (0.66, 1.54) |
| P for trend | 0.951 | 0.969 | 0.745 | 0.795 |

Model 1: adjusted for age, sex; Model 2: adjusted for heart rate, SBP, DBP, current smoker, current drinker, hyperlipidemia, Charlson comorbidity index, diabetes, and coronary heart disease based on model 1; Model 3: included variables in model 2 and further adjusted for uric acid, eGFR, cystatin C, TC, TG, LDL-C, HbA1c, FPG, Hcy, hsCRP, use of statins, use of aspirins, use of insulins, use of oral antidiabetic drugs, and antihypertensive drugs.

Abbreviations: SD, standard deviation; HR, hazard ratio; CI, confidence interval. Other abbreviations as presented in Table 1.

**Table S6**. Sensitivity analysis excluding subjects aged 80 and older.

| Exposure | Unadjusted | Model 1 | Model 2 | Model 3 |
| --- | --- | --- | --- | --- |
|  | HR (95% CI) | HR (95% CI) | HR (95% CI) | HR (95% CI) |
| **Total stroke** |  |  |  |  |
| Per SD increment | 1.27 (1.20, 1.35) | 1.27 (1.20, 1.35) | 1.30 (1.22, 1.38) | 1.34 (1.26, 1.43) |
| Quartiles |  |  |  |  |
| Q1 | Reference | Reference | Reference | Reference |
| Q2 | 0.95 (0.79, 1.16) | 0.97 (0.80, 1.17) | 0.96 (0.79, 1.16) | 0.97 (0.79, 1.17) |
| Q3 | 1.33 (1.11, 1.59) | 1.32 (1.11, 1.58) | 1.33 (1.11, 1.59) | 1.37 (1.14, 1.65) |
| Q4 | 1.64 (1.38, 1.94) | 1.67 (1.40, 1.98) | 1.74 (1.46, 2.08) | 1.84 (1.53, 2.21) |
| P for trend | <0.001 | <0.001 | <0.001 | <0.001 |
| **Ischemic stroke** |  |  |  |  |
| Per SD increment | 1.33 (1.24, 1.42) | 1.33 (1.25, 1.42) | 1.36 (1.27, 1.45) | 1.40 (1.31, 1.51) |
| Quartiles |  |  |  |  |
| Q1 | Reference | Reference | Reference | Reference |
| Q2 | 1.02 (0.82, 1.27) | 1.04 (0.83, 1.29) | 1.03 (0.83, 1.29) | 1.05 (0.84, 1.31) |
| Q3 | 1.50 (1.23, 1.84) | 1.49 (1.22, 1.83) | 1.50 (1.22, 1.84) | 1.56 (1.26, 1.92) |
| Q4 | 1.80 (1.48, 2.18) | 1.83 (1.51, 2.23) | 1.89 (1.55, 2.32) | 2.01 (1.63, 2.48) |
| P for trend | <0.001 | <0.001 | <0.001 | <0.001 |
| **Hemorrhagic stroke** |  |  |  |  |
| Per SD increment | 0.99 (0.88, 1.12) | 0.99 (0.88, 1.12) | 1.02 (0.90, 1.16) | 1.02 (0.89, 1.16) |
| Quartiles |  |  |  |  |
| Q1 | Reference | Reference | Reference | Reference |
| Q2 | 0.98 (0.70, 1.39) | 0.98 (0.69, 1.39) | 0.97 (0.68, 1.37) | 0.92 (0.65, 1.31) |
| Q3 | 1.15 (0.82, 1.60) | 1.13 (0.81, 1.58) | 1.15 (0.82, 1.61) | 1.13 (0.80, 1.60) |
| Q4 | 1.02 (0.73, 1.44) | 1.02 (0.72, 1.43) | 1.11 (0.78, 1.59) | 1.08 (0.75, 1.56) |
| P for trend | 0.692 | 0.712 | 0.382 | 0.444 |

Model 1: adjusted for age, sex; Model 2: adjusted for heart rate, SBP, DBP, current smoker, current drinker, hyperlipidemia, Charlson comorbidity index, and coronary heart disease based on model 1; Model 3: included variables in model 2 and further adjusted for uric acid, eGFR, cystatin C, TC, TG, LDL-C, HbA1c, FPG, Hcy, hsCRP, use of statins, use of aspirins, use of insulins, use of oral antidiabetic drugs, and antihypertensive drugs.

Abbreviations: SD, standard deviation; HR, hazard ratio; CI, confidence interval. Other abbreviations as presented in Table 1.

**Table S7**. Sensitivity analysis of excluding participants under treatment with glucose-lowering medications.

| Exposure | Unadjusted | Model 1 | Model 2 | Model 3 |
| --- | --- | --- | --- | --- |
|  | HR (95% CI) | HR (95% CI) | HR (95% CI) | HR (95% CI) |
| **Total stroke** |  |  |  |  |
| Per SD increment | 1.27 (1.16, 1.38) | 1.27 (1.17, 1.39) | 1.28 (1.19, 1.42) | 1.34 (1.22, 1.45) |
| Quartiles |  |  |  |  |
| Q1 | Reference | Reference | Reference | Reference |
| Q2 | 0.90 (0.71, 1.14) | 0.93 (0.74, 1.18) | 0.93 (0.73, 1.20) | 0.95 (0.72, 1.24) |
| Q3 | 1.26 (1.02, 1.58) | 1.28 (1.04, 1.61) | 1.27 (1.03, 1.60) | 1.28 (1.03, 1.63) |
| Q4 | 1.59 (1.27, 1.95) | 1.59 (1.24, 1.97) | 1.60 (1.26, 1.98) | 1.72 (1.35, 2.18) |
| P for trend | <0.001 | <0.001 | <0.001 | <0.001 |
| **Ischemic stroke** |  |  |  |  |
| Per SD increment | 1.37 (1.22, 1.49) | 1.36 (1.23, 1.50) | 1.39 (1.23, 1.52) | 1.42 (1.27, 1.58) |
| Categories |  |  |  |  |
| Quartiles |  |  |  |  |
| Q1 | Reference | Reference | Reference | Reference |
| Q2 | 0.90 (0.68, 1.17) | 0.92 (0.71, 1.22) | 0.93 (0.70, 1.24) | 0.94 (0.69, 1.28) |
| Q3 | 1.34 (1.06, 1.76) | 1.37 (1.08, 1.79) | 1.36 (1.06, 1.82) | 1.37 (1.06, 1.88) |
| Q4 | 1.69 (1.29, 2.28) | 1.74 (1.33, 2.26) | 1.77 (1.32, 2.24) | 1.81 (1.38, 2.33) |
| P for trend | <0.001 | <0.001 | <0.001 | <0.001 |
| **Hemorrhagic stroke** |  |  |  |  |
| Per SD increment | 1.01 (0.88, 1.27) | 1.01 (0.88, 1.27) | 1.03 (0.89, 1.30) | 1.02 (0.88, 1.29) |
| Quartiles |  |  |  |  |
| Q1 | Reference | Reference | Reference | Reference |
| Q2 | 1.09 (0.80, 1.78) | 1.10 (0.81, 1.80) | 1.12 (0.81, 1.82) | 1.10 (0.80, 1.81) |
| Q3 | 1.18 (0.86, 1.91) | 1.18 (0.86, 1.91) | 1.23 (0.88, 2.00) | 1.25 (0.89, 2.05) |
| Q4 | 1.18 (0.78, 1.99) | 1.18 (0.78, 2.00) | 1.25 (0.81, 2.11) | 1.22 (0.78, 2.10) |
| P for trend | 0.247 | 0.334 | 0.312 | 0.377 |

Model 1: adjusted for age, sex; Model 2: adjusted for heart rate, SBP, DBP, current smoker, current drinker, hyperlipidemia, Charlson comorbidity index, and coronary heart disease based on model 1; Model 3: included variables in model 2 and further adjusted for uric acid, eGFR, cystatin C, TC, TG, LDL-C, HbA1c, FPG, Hcy, hsCRP, use of statins, use of aspirins, and antihypertensive drugs.

Abbreviations: SD, standard deviation; HR, hazard ratio; CI, confidence interval. Other abbreviations as presented in Table 1.

**Table S8**. Sensitivity analysis of excluding participants who were receiving lipid-lowering therapy.

| Exposure | Unadjusted | Model 1 | Model 2 | Model 3 |
| --- | --- | --- | --- | --- |
|  | HR (95% CI) | HR (95% CI) | HR (95% CI) | HR (95% CI) |
| **Total stroke** |  |  |  |  |
| Per SD increment | 1.23 (1.19, 1.39) | 1.27 (1.19, 1.47) | 1.20 (1.22, 1.36) | 1.25 (1.26, 1.44) |
| Quartiles |  |  |  |  |
| Q1 | Reference | Reference | Reference | Reference |
| Q2 | 1.01 (0.82, 1.31) | 1.02 (0.84, 1.33) | 1.02 (0.83, 1.34) | 1.04 (0.85, 1.37) |
| Q3 | 1.35 (1.12, 1.72) | 1.34 (1.11, 1.71) | 1.36 (1.12, 1.74) | 1.42 (1.17, 1.82) |
| Q4 | 1.69 (1.40, 2.13) | 1.72 (1.42, 2.15) | 1.83 (1.50, 2.26) | 1.96 (1.60, 2.47) |
| P for trend | <0.001 | <0.001 | <0.001 | <0.001 |
| **Ischemic stroke** |  |  |  |  |
| Per SD increment | 1.33 (1.23, 1.53) | 1.32 (1.24, 1.51) | 1.35 (1.26, 1.55) | 1.41 (1.30, 1.62) |
| Quartiles |  |  |  |  |
| Q1 | Reference | Reference | Reference | Reference |
| Q2 | 1.02 (0.81, 1.28) | 1.04 (0.83, 1.30) | 1.04 (0.83, 1.30) | 1.07 (0.85, 1.35) |
| Q3 | 1.48 (1.21, 1.86) | 1.48 (1.20, 1.85) | 1.49 (1.21, 1.86) | 1.58 (1.27, 1.99) |
| Q4 | 1.83 (1.46, 2.26) | 1.86 (1.49, 2.32) | 1.93 (1.54, 2.40) | 2.11 (1.67, 2.79) |
| P for trend | <0.001 | <0.001 | <0.001 | <0.001 |
| **Hemorrhagic stroke** |  |  |  |  |
| Per SD increment | 1.01 (0.89, 1.22) | 1.01 (0.89, 1.24) | 1.04 (0.92, 1.29) | 1.04 (0.90, 1.28) |
| Quartiles |  |  |  |  |
| Q1 | Reference | Reference | Reference | Reference |
| Q2 | 1.15 (0.81, 1.83) | 1.15 (0.81, 1.83) | 1.15 (0.81, 1.82) | 1.10 (0.77, 1.73) |
| Q3 | 1.24 (0.84, 2.00) | 1.25 (0.82, 1.99) | 1.31 (0.88, 2.06) | 1.25 (0.83, 1.99) |
| Q4 | 1.12 (0.75, 1.88) | 1.12 (0.75, 1.89) | 1.16 (0.76, 1.98) | 1.18 (0.80, 2.08) |
| P for trend | 0.642 | 0.547 | 0.420 | 0.328 |

Model 1: adjusted for age, sex; Model 2: adjusted for heart rate, SBP, DBP, current smoker, current drinker, hyperlipidemia, Charlson comorbidity index, and coronary heart disease based on model 1; Model 3: included variables in model 2 and further adjusted for uric acid, eGFR, cystatin C, TC, TG, LDL-C, HbA1c, FPG, Hcy, hsCRP, use of aspirins, use of insulins, use of oral antidiabetic drugs, and antihypertensive drugs.

Abbreviations: SD, standard deviation; HR, hazard ratio; CI, confidence interval. Other abbreviations as presented in Table 1.

**Table S9**. Sensitivity analysis was conducted using the Fine-Gray competing risk model, considering non-stroke deaths as competing risk events.

| Exposure | Unadjusted | Model 1 | Model 2 | Model 3 |
| --- | --- | --- | --- | --- |
|  | SHR (95% CI) | SHR (95% CI) | SHR (95% CI) | SHR (95% CI) |
| **Total stroke** |  |  |  |  |
| Per SD increment | 1.22 (1.14, 1.30) | 1.22 (1.14, 1.30) | 1.24 (1.15, 1.32) | 1.29 (1.20, 1.39) |
| Quartiles |  |  |  |  |
| Q1 | Reference | Reference | Reference | Reference |
| Q2 | 0.93 (0.75, 1.16) | 0.96 (0.77, 1.19) | 0.97 (0.78, 1.20) | 0.99 (0.79, 1.23) |
| Q3 | 1.31 (1.07, 1.59) | 1.31 (1.07, 1.60) | 1.34 (1.09, 1.64) | 1.42 (1.16, 1.75) |
| Q4 | 1.52 (1.25, 1.84) | 1.54 (1.27, 1.87) | 1.60 (1.31, 1.95) | 1.77 (1.44, 2.17) |
| P for trend | <0.001 | <0.001 | <0.001 | <0.001 |
| **Ischemic stroke** |  |  |  |  |
| Per SD increment | 1.27 (1.18, 1.37) | 1.28 (1.18, 1.37) | 1.29 (1.19, 1.39) | 1.35 (1.25, 1.46) |
| Quartiles |  |  |  |  |
| Q1 | Reference | Reference | Reference | Reference |
| Q2 | 0.96 (0.75, 1.23) | 0.99 (0.77, 1.28) | 1.01 (0.78, 1.29) | 1.05 (0.81, 1.35) |
| Q3 | 1.53 (1.22, 1.91) | 1.53 (1.23, 1.92) | 1.56 (1.24, 1.96) | 1.67 (1.32, 2.10) |
| Q4 | 1.66 (1.33, 2.06) | 1.69 (1.35, 2.10) | 1.73 (1.38, 2.17) | 1.93 (1.52, 2.44) |
| P for trend | <0.001 | <0.001 | <0.001 | <0.001 |
| **Hemorrhagic stroke** |  |  |  |  |
| Per SD increment | 0.98 (0.85, 1.12) | 0.97 (0.85, 1.12) | 0.99 (0.86, 1.14) | 0.99 (0.85, 1.15) |
| Quartiles |  |  |  |  |
| Q1 | Reference | Reference | Reference | Reference |
| Q2 | 0.92 (0.62, 1.36) | 0.92 (0.62, 1.36) | 0.91 (0.61, 1.35) | 0.85 (0.57, 1.27) |
| Q3 | 0.99 (0.67, 1.45) | 0.98 (0.67, 1.44) | 1.02 (0.69, 1.50) | 1.01 (0.68, 1.51) |
| Q4 | 0.96 (0.65, 1.41) | 0.96 (0.66, 1.41) | 1.00 (0.67, 1.48) | 0.99 (0.65, 1.51) |
| P for trend | 0.938 | 0.935 | 0.880 | 0.834 |

Model 1: adjusted for age, sex; Model 2: adjusted for heart rate, SBP, DBP, current smoker, current drinker, hyperlipidemia, Charlson comorbidity index, and coronary heart disease based on model 1; Model 3: included variables in model 2 and further adjusted for uric acid, eGFR, cystatin C, TC, TG, LDL-C, HbA1c, FPG, Hcy, hsCRP, use of statins, use of aspirins, use of insulins, use of oral antidiabetic drugs, and antihypertensive drugs.

Abbreviations: SHR, subdistribution hazard ratio; SD, standard deviation; CI, confidence interval. Other abbreviations as presented in Table 1.

**Table S10**. A sensitivity analysis without adjustment for diabetes and hyperlipidemia was used.

| Exposure | Unadjusted | Model 1 | Model 2 | Model 3 |
| --- | --- | --- | --- | --- |
|  | HR (95% CI) | HR (95% CI) | HR (95% CI) | HR (95% CI) |
| **Total stroke** |  |  |  |  |
| Per SD increment | 1.26 (1.19, 1.33) | 1.26 (1.19, 1.34) | 1.30 (1.20, 1.38) | 1.34 (1.26, 1.44) |
| Quartiles |  |  |  |  |
| Q1 | Reference | Reference | Reference | Reference |
| Q2 | 0.95 (0.79, 1.15) | 0.97 (0.80, 1.18) | 0.97 (0.80, 1.18) | 0.98 (0.80, 1.19) |
| Q3 | 1.30 (1.09, 1.55) | 1.29 (1.08, 1.54) | 1.31 (1.09, 1.57) | 1.35 (1.12, 1.63) |
| Q4 | 1.60 (1.35, 1.90) | 1.63 (1.38, 1.93) | 1.73 (1.45, 2.06) | 1.84 (1.52, 2.20) |
| P for trend | <0.001 | <0.001 | <0.001 | <0.001 |
| **Ischemic stroke** |  |  |  |  |
| Per SD increment | 1.32 (1.23, 1.41) | 1.32 (1.24, 1.41) | 1.36 (1.27, 1.46) | 1.42 (1.31, 1.51) |
| Quartiles |  |  |  |  |
| Q1 | Reference | Reference | Reference | Reference |
| Q2 | 1.01 (0.81, 1.25) | 1.03 (0.83, 1.28) | 1.04 (0.84, 1.29) | 1.05 (0.85, 1.32) |
| Q3 | 1.47 (1.21, 1.79) | 1.46 (1.20, 1.78) | 1.48 (1.21, 1.82) | 1.53 (1.25, 1.89) |
| Q4 | 1.75 (1.45, 2.12) | 1.79 (1.48, 2.17) | 1.86 (1.54, 2.29) | 1.99 (1.60, 2.45) |
| P for trend | <0.001 | <0.001 | <0.001 | <0.001 |
| **Hemorrhagic stroke** |  |  |  |  |
| Per SD increment | 0.98 (0.87, 1.11) | 0.98 (0.87, 1.11) | 1.01 (0.89, 1.15) | 1.01 (0.89, 1.15) |
| Quartiles |  |  |  |  |
| Q1 | Reference | Reference | Reference | Reference |
| Q2 | 1.02 (0.73, 1.44) | 1.02 (0.73, 1.44) | 1.02 (0.73, 1.45) | 1.00 (0.69, 1.42) |
| Q3 | 1.13 (0.81, 1.57) | 1.12 (0.80, 1.56) | 1.12 (0.82, 1.59) | 1.10 (0.78, 1.56) |
| Q4 | 1.01 (0.72, 1.42) | 1.01 (0.72, 1.42) | 1.10 (0.78, 1.57) | 1.08 (0.76, 1.55) |
| P for trend | 0.817 | 0.835 | 0.435 | 0.446 |

Model 1: adjusted for age, sex; Model 2: adjusted for heart rate, SBP, DBP, current smoker, current drinker, Charlson comorbidity index, and coronary heart disease based on model 1; Model 3: included variables in model 2 and further adjusted for uric acid, eGFR, cystatin C, TC, TG, LDL-C, HbA1c, FPG, Hcy, hsCRP, use of statins, use of aspirins, use of insulins, use of oral antidiabetic drugs, and antihypertensive drugs.

**Abbreviations:** SD, standard deviation; HR, hazard ratio; CI, confidence interval. Other abbreviations as presented in Table 1.

**Table S11**. E-values for the observed associations between METS-IR and clinical outcomes.

|  | Total stroke | Ischemic stroke | Hemorrhagic stroke |
| --- | --- | --- | --- |
| Observed association* (per SD increment) | 1.33 (1.25, 1.42) | 1.39 (1.29, 1.49) | 1.01 (0.89, 1.15) |
| E-value for point estimate | 1.99 | 2.13 | 1.11 |
| E-value for confdence interval | 1.81 | 1.90 | 1.00 |

*The observed associations are the fully adjusted hazard ratios (95% confidence intervals) shown in Table 2 and are presented here for reference.

## 2.2 Supplementary Figures


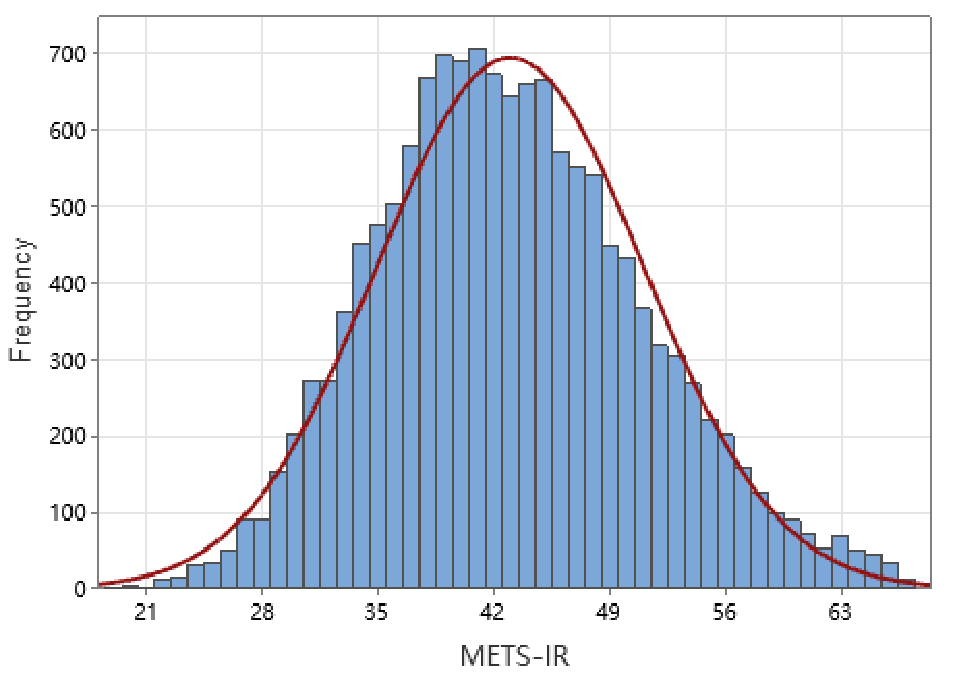


**Figure S1** Histograms show the population distribution of the METS-IR.
